# Why do healthcare professionals fail to escalate as per the early warning system (EWS) protocol? A qualitative evidence synthesis of the barriers and facilitators of escalation

**DOI:** 10.1186/s12873-021-00403-9

**Published:** 2021-01-28

**Authors:** S. M. O’Neill, B. Clyne, M. Bell, A. Casey, B. Leen, S. M. Smith, M. Ryan, M. O’Neill

**Affiliations:** 1 The Health Information and Quality Authority (HIQA), City Gate, Mahon, Cork, T12 Y2XT Ireland; 2 HRB Centre for Primary Care Research and Department of General Practice, Royal College of Surgeons in Ireland, Dublin, Ireland; 3 The Deteriorating Patient Recognition and Response Improvement Programme (DPIP), Clinical Design and Innovation, Health Service Executive, Dr. Steeven’s Hospital, Steevens’ Lane, D08W2A8 Dublin, Ireland; 4 Regional Librarian, Health Service Executive South, Kilkenny, Ireland

**Keywords:** Early warning system, Failure to escalate, Thematic analysis, Barriers, Facilitators

## Abstract

**Background:**

Early warning systems (EWSs) are used to assist clinical judgment in the detection of acute deterioration to avoid or reduce adverse events including unanticipated cardiopulmonary arrest, admission to the intensive care unit and death. Sometimes healthcare professionals (HCPs) do not trigger the alarm and escalate for help according to the EWS protocol and it is unclear why this is the case. The aim of this qualitative evidence synthesis was to answer the question ‘why do HCPs fail to escalate care according to EWS protocols?’ The findings will inform the update of the National Clinical Effectiveness Committee (NCEC) National Clinical Guideline No. 1 Irish National Early Warning System (INEWS).

**Methods:**

A systematic search of the published and grey literature was conducted (until February 2018). Data extraction and quality appraisal were conducted by two reviewers independently using standardised data extraction forms and quality appraisal tools. A thematic synthesis was conducted by two reviewers of the qualitative studies included and categorised into the barriers and facilitators of escalation. GRADE CERQual was used to assess the certainty of the evidence.

**Results:**

Eighteen studies incorporating a variety of HCPs across seven countries were included. The barriers and facilitators to the escalation of care according to EWS protocols were developed into five overarching themes: Governance, Rapid Response Team (RRT) Response, Professional Boundaries, Clinical Experience, and EWS parameters. Barriers to escalation included: Lack of Standardisation, Resources, Lack of accountability, RRT behaviours, Fear, Hierarchy, Increased Conflict, Over confidence, Lack of confidence, and Patient variability. Facilitators included: Accountability, Standardisation, Resources, RRT behaviours, Expertise, Additional support, License to escalate, Bridge across boundaries, Clinical confidence, empowerment, Clinical judgment, and a tool for detecting deterioration. These are all individual yet inter-related barriers and facilitators to escalation.

**Conclusions:**

The findings of this qualitative evidence synthesis provide insight into the real world experience of HCPs when using EWSs. This in turn has the potential to inform policy-makers and HCPs as well as hospital management about emergency response system-related issues in practice and the changes needed to address barriers and facilitators and improve patient safety and quality of care.

**Supplementary Information:**

The online version contains supplementary material available at 10.1186/s12873-021-00403-9.

## Background

Acute physiological deterioration is a time-crucial medical emergency and failure to detect and treat patient deterioration in a timely manner poses a threat to patient safety, which may lead to adverse patient outcomes [1, 2]. Deterioration of a patient’s condition in hospital is frequently preceded by measurable physiological abnormalities. Regular measurement, documentation and interpretation of vital signs and other physiological observations is an essential requirement for recognising clinical deterioration [3]. Early recognition of clinical deterioration, followed by prompt and effective action, can minimise the occurrence of adverse events such as unanticipated cardiopulmonary arrest, [4] and may mean that a lower level of intervention, and thus resources, is required to stabilise a patient.

Healthcare organisations adopt a multi-faceted approach to the detection and management of deteriorating patients. Approaches include early warning systems (EWSs) which incorporate the recognition, escalation, response and clinical governance of the deteriorating patient; targeted education programmes for healthcare professionals, and standardised approaches to clinical and interdepartmental handover [5]. One element of an EWS is the track and trigger tool, incorporated into the patient observation chart. The track and trigger tool categorises a patient’s severity of illness and prompts escalation of care according to a pre-agreed protocol as appropriate. The EWS thus alerts the health care professional (HCP) who in turn (if the protocol deems it necessary), should escalate care and/or summon or trigger the Rapid Response Team (RRT). The track and trigger tool is used to assist, rather than over-ride HCPs clinical judgement and decision-making.

Previous research has raised questions about the overall effectiveness of EWSs and RRTs, [6, 7], however minimal research has focused on how EWSs and RRTs are viewed by the HCPss who use them in practice. Studies have shown that even though a number of serious adverse events have occurred, in very few instances (8% in one study) [8] was the escalation protocol strictly adhered to [8–10]. Why this is the case is vital information for national level guidance and planning, including for hospital managers to more fully understand the implications of EWS and RRT implementation for those who use them in practice every day. The aim of this systematic review was to answer “why do HCPs fail to escalate as per EWS protocols?” It was conducted by members of the Health Research Board-funded Collaboration in Ireland for Clinical Effectiveness Reviews team (HRB-CICER) in the Health Information and Quality Authority (HIQA) of Ireland as part of a series of systematic reviews which arose directly from six questions posed by members of the Irish National Early Warning System (INEWS) Guideline Development Group, who have updated the INEWS National Clinical Guideline (No 1.) recently (see INEWS Guideline Version 2) endorsed by the National Clinical Effectiveness Committee (NCEC). Hence, the findings of this review will inform the Irish national health service response to acute physiological deterioration in adult in-patients and the use of EWSs in the acute hospital setting in Ireland.

## Methods

In reporting this qualitative evidence synthesis we have adhered to the ENTREQ (Enhancing transparency in reporting the synthesis of qualitative research) guidelines [11].

### Protocol registration

The protocol for this systematic review has been registered on the PROSPERO database of systematic reviews and meta-analyses (Link: http://www.crd.york.ac.uk/PROSPERO/display_record.php?ID=CRD42018088048).

### National Clinical Guideline Update

This qualitative evidence synthesis was conducted to inform the update of the Irish National Clinical Guideline No. 1 INEWS (for which six review questions were conducted in total). The six review questions were as follows:

1. What EWSs and or track and trigger systems are currently in use for the detection or timely identification of physiological deterioration in adult (non-pregnant) patients in acute health care settings?
2. How effective are the different EWSs in terms of improving key patient outcomes in adult (non-pregnant) patients in acute health care settings?
3. What education programmes have been established to train healthcare professionals (HCPs) relating to the implementation of EWSs or track and trigger systems for the detection/timely identification of physiological deterioration in adult (non-pregnant) patients in acute health care settings?
4. What are the findings from the economic literature on cost-effectiveness, cost impact and resources involved with the implementation of EWSs or track and trigger systems for the detection or timely identification of physiological deterioration in adult (non-pregnant) patients in acute health care settings?
5. Are modified EWSs (e.g. CREWS) more effective than the NEWS for the detection or timely identification of physiological deterioration in specific adult sub-populations in acute health care settings?
6. Why do HCPs fail to escalate as per the EWS escalation protocol?

### Aim

To systematically review the qualitative literature which addresses the question as to why HCPs may fail to escalate as per the EWS protocols and to identify barriers and facilitators to escalation (review question 6 only).

### Search strategy

We searched for potentially eligible studies published between January 2011 until February 19th 2018 in 11 electronic databases including CINAHL, Medline and Embase (See additional File 1 for the full list of electronic databases searched) as well as five grey literature databases including OpenGrey System and Open University Dedicated Grey Literature and more than 30 relevant websites (see Additional File 1 for the full list of grey literature resources searched). A combination of EWS specific search terms (e.g. “early warning” OR “warning system” OR “warning scor*” OR “failure to rescue”) and escalation search terms (e.g. *“Failure to escalate” OR “fail to escalate” OR “non-adherence to EWS escalation protocol”)* were used in the search (see Additional File 2 for the full list of search terms used) with the assistance of a librarian. Limitations applied included a date restriction (January 2011 until February 19th 2018) and Language (English language studies only).

### Inclusion and exclusion criteria

We included studies which investigated why HCPs (including doctors, nurses, allied health professionals, etc.) may fail to escalate as per the EWS protocols which included participants from an adult acute setting only, based in a high or very high HDI country. Studies including participants from obstetric, paediatric and emergency settings were excluded. We only included studies with a qualitative study design (e.g. focus group or individual interviews, observation, document analysis) that used qualitative methods of analysis (i.e. thematic analysis, framework analysis, grounded theory). Qualitative studies (e.g. open-ended survey questions) where the responses were analysed using descriptive statistics were excluded.

### Screening

The results of the systematic search were exported into Endnote reference manager and duplicates were removed. Two reviewers initially screened titles and abstracts to remove any irrelevant studies. The full texts of potentially eligible studies were obtained and screened by two reviewers applying the inclusion and exclusion criteria. Disagreements were resolved by discussion, or if necessary, a third reviewer.

### Results of overall search

The search strategy for all six review questions identified 54,271 potentially relevant records through searching the listed electronic databases and grey literature sources. After removing duplicates, 36,445 records were screened independently by two reviewers, with a further 36,110 references excluded based on titles and abstracts. A total of 335 full-text articles were assessed for eligibility. Of these, 203 references were excluded according to the inclusion and exclusion criteria. This resulted in 132 studies being included in the overall review (for the six questions) to inform the NCG. Manual checking of the reference lists of included studies identified a further 22 eligible studies, bringing the total number of studies to 154 (for the six questions).

### Data extraction and quality appraisal

The following data were extracted by two reviewers independently using standardised data extraction forms: study author, country, study setting, study design, qualitative methodology, type of healthcare professional, type of EWS or emergency response system in operation and the key findings of each study (i.e. barriers and facilitators to escalation of the EWS). Quality appraisal of the included studies was completed by two reviewers independently using the Critical Appraisal Skills Programme (CASP) checklist for qualitative studies, [12] which is a set of 10 questions to address the rigour of the study.

### Certainty of the evidence

The GRADE-CERQual (Confidence in the Evidence from Reviews of Qualitative research) approach was used to summarise confidence in the evidence [13]. Four components contribute to an assessment of confidence in the evidence for an individual review finding: methodological limitations, relevance, coherence, and adequacy of data. Confidence in the evidence was graded by two reviewers as high, moderate, low, or very low for each key finding.

### Data synthesis

The evidence on why HCPs may fail to escalate was synthesised in the form of a thematic analysis [14, 15]. Two reviewers (a Health Services Researcher with a background in epidemiology, and a Research Lecturer with a background in social science and sociology of health and illness) read all included papers a number of times to achieve absorption of the data. Both reviewers manually extracted the text from each study (results section only) and coded line by line in Excel, and developed initial sub-themes and overarching themes independently. Following multiple discussions and re-analysis of the draft themes and sub-themes as well as presentation of the findings to the GDG, the reviewers reached consensus on the final overarching themes and sub-themes. The findings are presented according to themes generated which were coded for each included study according to barriers and facilitators of escalation.

## Results

**Fig. 1:**
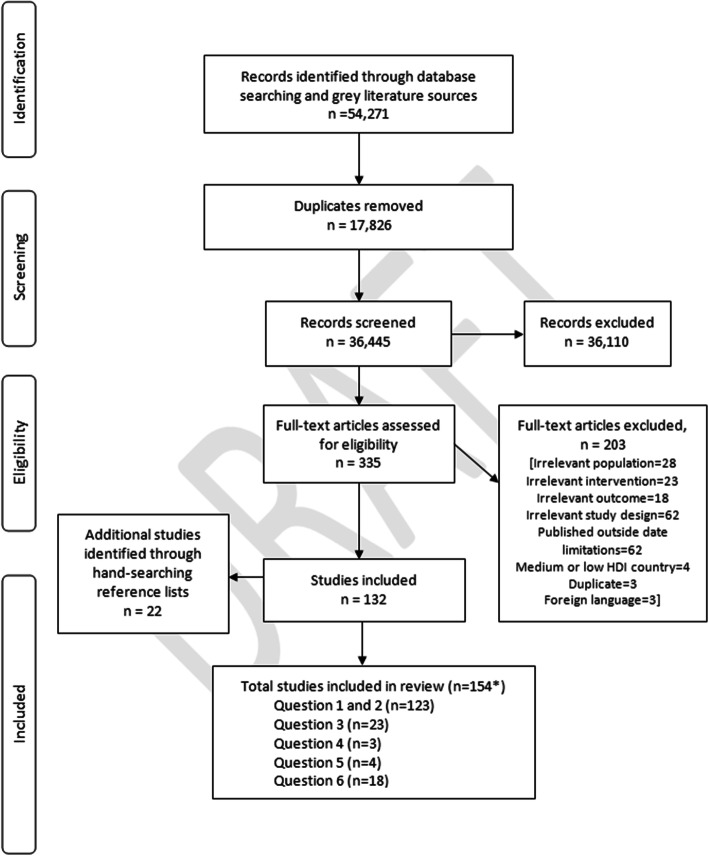
Study flow diagram of the systematic search. The systematic search was conducted for six review questions. These include: 1: What EWSs and or track and trigger systems are currently in use for the detection or timely identification of physiological deterioration in adult (non-pregnant) patients in acute health care settings? 2: How effective are the different EWSs in terms of improving key patient outcomes in adult (non-pregnant) patients in acute health care settings? 3: What education programmes have been established to train healthcare professionals (HCPs) relating to the implementation of EWSs or track and trigger systems for the detection/timely identification of physiological deterioration in adult (non-pregnant) patients in acute health care settings? 4: What are the findings from the economic literature on cost-effectiveness, cost impact and resources involved with the implementation of EWSs or track and trigger systems for the detection or timely identification of physiological deterioration in adult (non-pregnant) patients in acute health care settings? 5: Are modified EWSs (e.g. CREWS) more effective than the NEWS for the detection or timely identification of physiological deterioration in specific adult sub-populations in acute health care settings? 6: Why do HCPs fail to escalate as per the NEWS escalation protocol? * = Note the total number of eligible studies is (*n* = 154). However the total across all six review questions equals more than n = 154 as some studies were eligible for more than one review question

For this qualitative evidence synthesis, there were 18 eligible studies, which focused on why HCPs may fail to escalate as per the EWS protocols (see Fig. 1, Study Flow Diagram). The findings of these 18 qualitative studies are synthesised and presented in this paper.

### Characteristics of included studies

Eighteen qualitative studies were eligible for inclusion with three conducted in Australia, [16–18] six in the UK, [19–24] five in the USA, [25–29] and one each in Ireland, [30] Norway, [31] Denmark, [9] and Singapore [32]. Ten studies included nurses only (registered, unregistered), [9, 18, 22, 24, 25, 27–29, 31, 32] three studies includes nurses and doctors only, [17, 23, 30] and five studies included a mixture of HCPs and staff [nurses, physicians, administrators, respiratory technicians, health care assistants, safety leads and managers] [16, 19–21, 26]. A total of 599 participants were interviewed across the studies with sample sizes ranging from six participants [24] to 218 participants [16]. To gain an understanding of the barriers and facilitators to escalation, eight studies used face-to-face interviews, [18, 19, 22, 23, 25, 26, 30, 32] and seven studies used focus groups [9, 16, 17, 24, 27, 28, 31]. Three studies [20, 21, 29] used a combination of methods including interviews, observations of interactions, and documentary evidence [protocols and audit data], two of which were conducted in the same hospital and sample [20, 21]. The first study by Mackintosh (2012) [20] contained 150 h of observations and used thematic analysis while the second study (Mackintosh, 2014) [21] contained 180 h of observation and the analysis focused on the structural conditions that shape delivery of the rapid response drawing on Bourdieu’s logic of practice. Data from both were extracted for this thematic analysis. The key study characteristics are outlined in Table 1.

**Table 1:** Why do healthcare professionals fail to escalate as per the EWS protocol? Study Characteristics

Author (year), Country Study setting	Study design (focus group interviews, face-to-face interviews, other) Qualitative methodology (e.g. Ethnography, narrative, phenomenological, grounded theory)	Type of healthcare professional	Outcomes assessed: Data describing the views, experiences and behaviours of HCPs and why there is a failure to escalate as per protocol with EWS	Type of EWS or RRT in operation
Astroth (2012), [25] USA 3 medical/surgical units, community hospital	Face-to-face interviews Analysis: concept analysis	Nurses (*n* = 15)	Facilitators and barriers to RRT activation	RRT in a 155-bed Midwestern community hospital. No other details provided.
Benin (2012), [26] USA 1 academic hospital	Face-to-face interviews Analysis: thematic analysis and the constant comparative method	49 participants: Nurses [18], primary team senior attending physicians [6], house staff members [6] RRT attending physicians [4], RRT critical care nurses [4], RRT respiratory technicians [3] administrators [8]	To create a comprehensive view of the impact and value of an RRT on a hospital and its staff, the objective of this study was to qualitatively describe the experiences of and attitudes held by nurses, physicians, administrators, and staff regarding RRTs.	Adult RRT implemented in 2005 consisting of a hospitalist physician, a critical care nurse and a respiratory therapist. The RRT was triggered by specific criteria which were not listed in the study.
Braaten (2015), [29] USA Non-teaching, acute care hospital	Cognitive work analysis. Face-to-face interviews, Document review Analysis: Directed content analysis	Nurses (n = 12) 11 female, 1 male from the medical-surgical wards	To describe factors within the hospital system that shape medical-surgical nurses RRT activation behaviour	Conducted in the medical-surgical units in a large hospital in Colorado with a well-established RRT system with a standardised policy and calling criteria, developed and implemented in 2005.
Cherry (2015), [24] UK Acute NHS hospital	Focus groups Analysis: Framework analysis technique	Nurses (*n* = 6) 1 focus group 1 band 7, 1 band 6 and 4 staff nurses from the AMU	To understand the attitudes of qualified nursing staff on the AMU concerning the MEWS score chart used to monitor patients.	The MEWS was in use in the AMU and the hospital, including 8 parameters (respiratory rate, oxygen saturation, inspired oxygen, heart rate, systolic blood pressure, central nervous system level using the alert, voice, pain, unresponsive (AVPU) tool, urine output and temperature. Observations were to be measured minimum 12-hourly and more frequently depending on the MEWS score.
Chua (2013), [32] Singapore 1 acute hospital	Face-to-face interviews with critical incident technique (CIT) Analysis: content analysis	Enrolled nurses (ENs) (n = 15) ENs: non-registered nursing staff provide bedside nursing care and routine vital signs monitoring and convey findings to the registered nurses	Experiences of ENs with the deteriorating patient in pre-cardiac arrest situations. Strategies to enhance the role of ENs in detecting and managing ward deteriorating patients	No system reported but vital signs were used to detect deterioration.
Elliott (2015), [16] Australia 8 different hospital sites	Focus groups (44) Analysis: thematic analysis	Staff (*n* = 218) (mainly nurses and doctors)	Experiences and views of staff using ORCs in clinical practice	ORCs based on the ADDS and a RRT with clear protocols for escalation.
Johnston (2014), [19] UK 3 hospitals across London	Semi-structured interviews Analysis: Emergent theme analysis	41 participants: attending/senior resident grade surgeons [16], surgical postgraduate year 1 (11), surgical nurses [6], intensivists [4], critical care outreach team members [4]	The current escalation landscape; When junior doctors and nurses should escalate care; Information required prior to senior review; Barriers to successful escalation of care; Strategies to improve the escalation process.	Escalation of care across the surgical pathway from the specialities of General Surgery, Vascular Surgery, and Urology from 3 London hospitals was examined. No other details provided.
Kitto (2015), [17] Australia 4 hospitals	Multiple case study (focus groups) Conceptual framework: Collective competence and inter-professional conceptual framework Analysis: Directed content analysis & conventional content analysis	89 participants (10 focus groups): doctors [27], nurses (62)	Medical and nursing staff experiences of RRT Explore the reasons why staff members do not activate the RRT	RRT in 4 different hospitals. No other details provided.
Lydon (2016), [30] Ireland 1 teaching hospital	Mixed Methods, semi-structured interviews Analysis: Deductive content analysis	30 participants: Interns [1st year of postgraduate training] [18], Senior NCHDs [2], Nurses [10]	To examine the perceptions of a national PTTS among nurses and doctors and to identify the variables that impact on intention to comply with protocol.	A PTTS using the NEWS and ISBAR communication tool
Mackintosh (2012), [20] UK 2 tertiary teaching hospitals **Same sample as Mackintosh (2014)	Ethnography; Observation of interactions among multi-professional healthcare staff and patient management processes; semi-structured interviews. Analysis: framework approach	150 h of observations 35 interviews: Doctors [14], Ward and critical care nurses [11], Healthcare assistants [4], Safety leads and managers [6]	To illuminate the different contextual processes which contribute to patients’ rescue trajectories and clarify the benefits and limitations of particular safety strategies within a pathway of care for the acutely ill patient.	Five strategies were in use across 2 hospitals. At Westward, an EWS, escalation protocol, communication protocol (SBAR) and CCOT (comprised of nurses, physiotherapists and intensive care physicians) were in operation. In Eastward, there was an EWS and 2 of the medical wards were piloting an intelligent assessment technology (IAT) which utilised a different scoring system to the EWS already in use in Westward and included a personal digital assistant (PDA).
Mackintosh (2014), [21] UK 2 tertiary teaching hospitals **Same sample as Mackintosh (2012)	Ethnography: - Observations - Documentary evidence- protocols and audit data - Semi-structured interviews Theoretical framework: Bourdieu - logic of practice	180 h of observations: Interactions between health care staff, recording of patients’ vital signs, ward rounds, handovers and multi-disciplinary team meetings. 35 interviews: health care assistants, nurses, physicians, critical care staff and managers	Interviews with staff focused on the management of escalation of care, the role of the RRT, and the influence of organisational contextual factors on its application.	
Massey (2014), [18] Australia 1 public teaching hospital	In-depth semi structured interviews.	Registered ward nurses (*n* = 15)	Nurses’ experiences and perceptions of using and activating METs	A large public teaching hospital with a well-established MET, using a single parameter system with specific MET calling criteria based on vital sign observations and thresholds.
McDonnell (2013), [22] UK District general hospital	Mixed methods with semi-structured interviews. Interviews before the training and approximately 6 weeks after the introduction of new charts Analysis: thematic framework	Nurses (n = 15)	Knowledge and confidence of nursing staff in an acute hospital	A 2 tier track and trigger system using either the standard observation chart or the detailed Patient at Risk (PAR) chart. Patients could be stepped up to the PAR chart (if they triggered) or stepped down to the standard chart. A CCOT was also in place.
Pattison (2012), [23] UK Specialist hospital	Grounded theory principles. Interviews. Analysis: Constant comparative technique.	9 participants: Nurses [7], Doctors [2]	To explore referrals to CCOT, associated factors around patient management and survival to discharge, and the qualitative exploration of referral characteristics (identifying any areas for service improvement around CCOT).	MEWS and CCOT in a specialist hospital.
Petersen (2017), [9] Denmark University hospital	Focus groups Analysis: Content analysis	Nurses (*n* = 18) 5 focus groups (3–5 participants in each) (2 male, 16 female from the medical and surgical acute care wards)	To identify barriers and facilitators related to three aspects of the EWS protocol: 1) adherence to monitoring frequency; 2) informing doctors of patients with an elevated EWS (≥3), and 3) call for the MET	A modified version of the NEWS has been in use in hospitals in the Capital Region of Denmark since 2013. Parameters included: respiratory rate, oxygen saturation, supplemental oxygen, temperature, systolic blood pressure, heart rate, and level of consciousness. Clear protocol for action based on EWS trigger scores in operation.
Stafseth (2016), [31] Norway University hospital	Semi-structured focus group interviews Analysis: Thematic analysis	Nurses (*n* = 7) 2 focus groups of 3 and 4 nurses.	Registered nurses’ experiences with the early detection and recognition of vital function failures and experiences with the use of the MEWS and the MICN.	A track and trigger system comprised of the MEWS and a 24-h on-call MICU, which was a nurse-led support service (not a team). MICU nurses were registered nurses with two years postgraduate education in critical care nursing and extensive experience in critical care.
Stewart (2014), [27] USA Acute care hospital	Mixed-methods; Focus groups Analysis: Thematic analysis	Nurses (*n* = 11) 5 focus groups with between 1 and 4 attendees, providers.	Perceptions of barriers and facilitators to the use of MEWS at the bedside	The MEWS scoring system was implemented in the hospitals electronic medical record system in 2011 where a RRT also exists.
Williams (2011), [28] USA Community hospital	Focus groups Analysis: Content analysis	Nurses (*n* = 14) 6 focus groups Staff nurses [6], Nurse clinicians [2] Supervisor/educators [6]	Thoughts and feelings about shared and “lived” experiences surrounding RRT use.	156-bed community hospital with a nurse-led RRT implemented in 2005. RRT consisted of an ICU registered nurses, an emergency department registered nurse and a respiratory therapist. Hospitalists often responded to RRT calls but were not obliged to according to hospital protocol.

Legend: **ADDS:** Adult Deterioration Detection System; **AMU:** Acute Medical Unit; **CCOT**: Critical Care Outreach Team; **CIT:** Critical Incident Technique; **EN**: Enrolled Nurses; **EWS**: Early Warning System; **HCP**: Health Care Professional; **MEWS**: Modified Early Warning System; **MET:** Medical Emergency Teams; **MICN**: Mobile Intensive Care Nurse; **NCHD**: Non Consultant Hospital Doctor; **ORC**: Observation Response Chart; **PTTS**: Physiological Track and Trigger System; **RRT**: Rapid Response System; **RRT**: Rapid Response Team

**Fig. 2:**
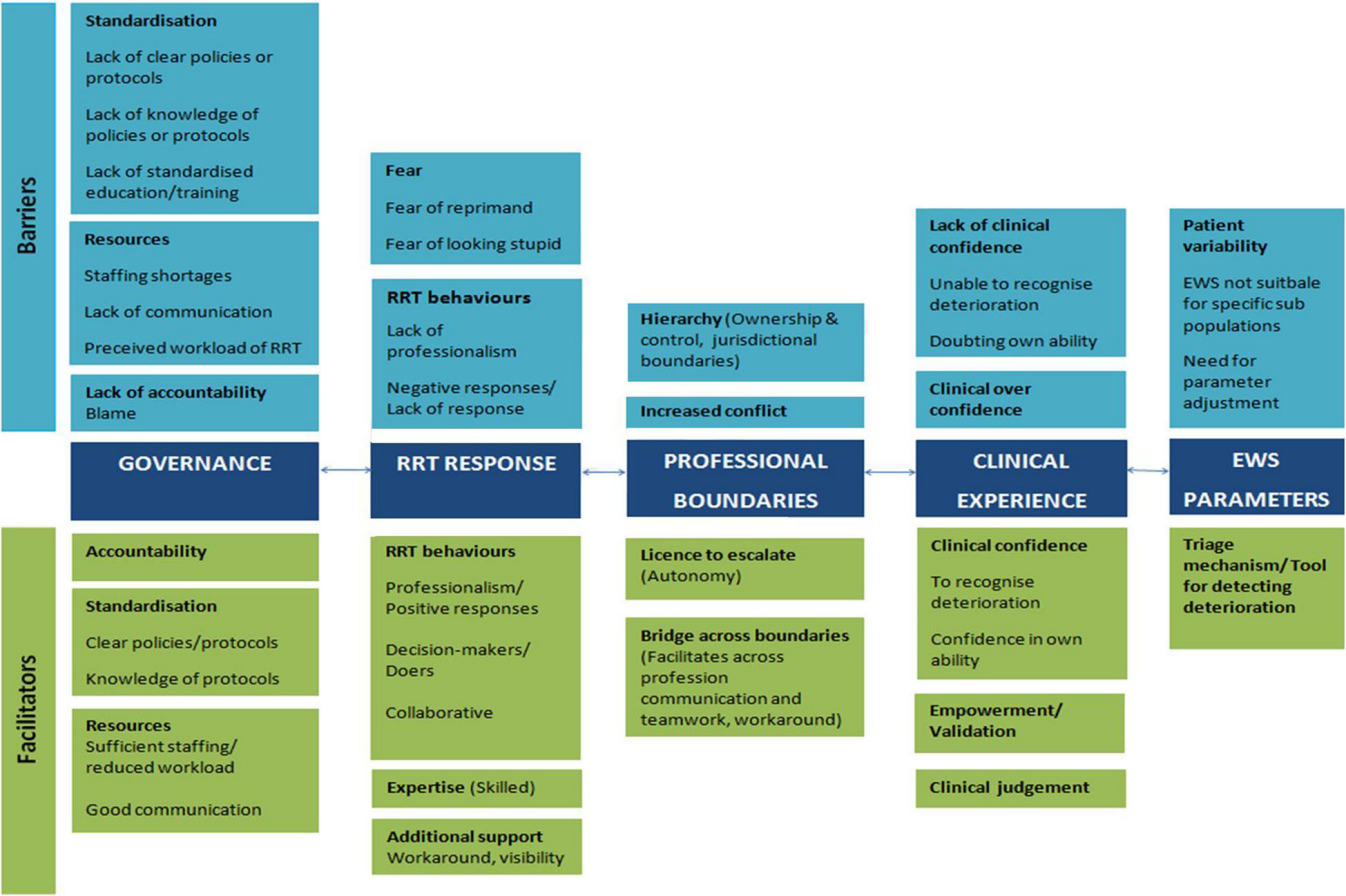
Schematic of study themes and sub-themes identified in the evidence synthesis. The overarching key themes (*n* = 5) identified as ‘barriers and facilitators’ of escalation are presented in dark blue. The sub-themes (*n* = 10) are presented in light blue for the ‘barriers’ to escalation and green (*n* = 12) for the ‘facilitators’ to escalation

Thematic synthesis produced five overarching themes and 22 sub-themes with multiple interdependencies. These are categorised into barriers (twelve sub-themes) and facilitators (ten sub-themes) of escalation. These are described for each of the five overarching themes: Governance, Rapid Response Team (RRT) Response, Professional Boundaries, Clinical Experience, and EWS Parameters (see Fig. 2).

### Synthesis of results

#### Barriers to escalation

Quotations from either primary study participants (*in italics*) or study authors relating to the ‘barriers’ for each key theme (*n* = 5) and sub-theme (*n* = 10) are presented in Table 2.

**Table 2:** Key themes of the barriers of escalation amongst healthcare professionals

Key Themes	Sub-themes	Characteristics of studies from which sub-themes were derived: Type of participant and setting	Illustrative quotations (*Italicised text* = primary quote from a study participant; non-italicised text = secondary quote from study authors)
**Governance**	**Lack of accountability** [20, 21, 32]	Enrolled nurses (non-registered nurses who assist registered nurses) in 1 Singaporean hospital [32]; HCAs, nurses, physicians, critical care staff and managers in 2 UK hospitals [20, 21]	A few participants strongly reiterated the need for some form of nursing documentation which specified that they had informed the RN-in-charge of patient deterioration. This was to safeguard the ENs from being blamed for not reporting patient deterioration: *“The EN should have charting and documentation that indicates this staff nurse had been informed*. *.. so then at least we know that we’re safe and we don’t get into trouble. (P3)”* [32]
	**Standardisation** -Lack of clear policies/protocols [19, 20, 24, 29] -Lack of knowledge of policies/protocols [16–19, 25, 29] -Lack of standardised education/training [17, 18, 24, 25, 30, 32]	HCAs, nurses, physicians, critical care staff and managers in 2 UK hospitals [20]; Senior resident surgeons, surgical postgraduates year 1, intensivists, and critical care outreach team members from 3 UK hospitals [19]; Nurses in 1 US hospital [25]; Mainly doctors and nurses in 8 Australian hospitals [16]; Doctors and nurses in 4 Australian hospitals [17]; Nurses in 1 Australian hospital [18]; Enrolled nurses (non-registered nurses who assist registered nurses) in 1 Singaporean hospital [32]; Year 1 interns, Senior NCHDs and nurses in 1 Irish hospital [30]; Nurses in 1 US hospital [29]; Nurses in 1 UK hospital [24]	“*On a number of occasions I’ve had difficulties contacting a senior because there is no fixed framework for doing so”* [19]. “*Maybe if we had a clearer-cut criteria on when we do call an RRT and when we wait. You know?. .. People aren’t sure. Do we wait until they get this bad. .. or their O*_*2*_ *requirements are at this level? At what point do we need to call them?.*. *.”* [29] *“I think it’s probably a lack of understanding of the MET and how it should be used. People don’t see it as an early intervention thing; I am not sure how you go about changing that. I can see that the patient is deteriorating and I can see that poor decisions are being made and it’s very frustrating, yet a MET is not called because the patient is not sick enough for a MET; it’s amazing”* [18]. A few participants stated they had not received any education other than when the RRT was first developed. One nurse indicated she had not attended any RRT educational sessions [25].
	**Resources** -Staffing shortages [9, 16, 19, 23, 26, 29, 30, 32] -Poor communication/use of handover tools [16, 19, 30] -Perceived workload of RRT [16, 19, 23, 25, 30, 32]	HCPs from 1 US hospital [26]; Mainly doctors and nurses in 8 Australian hospitals [16]; Year 1 interns, Senior NCHDs and nurses in 1 Irish hospital [30]; Enrolled nurses (non-registered nurses who assist registered nurses) in 1 Singaporean hospital [32] Senior resident surgeons, surgical postgraduates year 1, intensivists, and critical care outreach team members from 3 UK hospitals [19]; Nurses and doctors from 1 UK hospital [23]; Nurses in 1 US hospital [25]	*“Adherence to the NEWS protocol was impaired or impossible due to insufficient staffing levels...”* [33] Communicating actions recommended by the chart to escalate care was also sometimes challenging for participants, especially when attempting to obtain a response from medical officers [16]. Perceived busyness of the ICU nurses discouraged participants from RRT activation. Participants noted that responding RRT members occasionally talked about how busy they were [25].
**RRT Response**	**RRT Behaviours** - Lack of professionalism [9, 17–19, 25, 28, 29, 31] -Negative response/Lack of response [9, 17–19, 24, 25, 28, 31]	Nurses in 1 US hospital [25]; HCPs in 3 UK hospitals [19]; Doctors and nurses in 4 Australian hospitals [17]; Nurses in 1 Australian hospital [18]; Nurses in 1 Norwegian hospital [31]; Nurses in 1 US hospital [28]	*“They don’t want to listen to our side of the story or what we have to say. They are just more like, “I’m in charge and this is what you have to do,” so they’re more like barking out orders rather than kind of flowing with whatever we’ve already been doing and working as a team...”* [29] Sometimes team members complained about the need for the RRT call: “*Why did you call? This wasn’t necessary”. “Once a nurse gets attitude (from RRT members), they don’t want to call again*” [25].
	**Fear** -Fear of reprimand [9, 17–19, 29]	Nurses in 1 US hospital [25]; HCPs in 1 US hospital [26]; HCPs in 3 UK hospitals [19]; Doctors and nurses in 4 Australian hospitals [17]; Nurses in 1 Australian hospital [18].	*“Nurses feel like they are going to be told off for wasting the medical emergency team’s time. Even though worried or concerned is on the little cards that we all carry around. That message has not been embraced by the nursing staff because people are still frightened I think. Talking to people they still think they are going to get told off or there are going to be repercussions.” (Mary)* [34].
	-Fear of looking stupid [17–19, 25, 26]		This theme is understood as either refusing to activate a MET or pausing before activating a MET. Participants said*, “I don’t know if it would be the right thing to do”, “I don’t want to look like an idiot”* [18].
**Professional Boundaries**	**Increased Conflict** [17, 18, 20, 23, 25, 26, 30]	Nurses and doctors from 1 UK hospital [23]; Doctors and nurses in 4 Australian hospitals [17]; Nurses in 1 Australian hospital [18]; Year 1 interns, Senior NCHDs and nurses in 1 Irish hospital [30]; HCAs, nurses, physicians, critical care staff and managers in 2 UK hospitals [20]; Nurses in 1 US hospital; (25) HCPs in 1 US hospital [26]	RRT improved morale between nurses and RRT but increased conflict between nurses and physicians [26]. Interns frequently cite the NEWS as a source of conflict between doctors and nurses. For example an intern commented that: “*some nurses see NEWS as something where they bring you and then wash their hands - they’re rung someone, anyone, so their job is now done*” (Intern 5) [30]
	**Hierarchy** (ownership and control, jurisdictional boundaries) [17, 19–21, 24–26, 29]	Doctors & nurses in 4 Australian hospitals [17]; HCPs in 3 UK hospitals [19]; HCAs, nurses, physicians, critical care staff & managers in 2 UK hospitals [20]; Nurses in 1 US hospital [25]; HCPs in 1 US hospital [21, 26]	*“Sometimes they* [primary ward physician]*….have a bit of an attitude thing, oh I can handle this. This is my patient. I know this patient. I didn’t want a rapid response to be called. You know we get a fair amount of that, but not as much as we did in the beginning. In the beginning....nurses were being yelled at by the primary team....how dare you call a rapid response on my patient... they seem to be more receptive now [SWAT nurse]”* [26].
**Clinical Experience**	**Clinical over confidence** [9, 19, 23, 25, 29] **Lack of clinical confidence** [18, 25, 29] -Unable to recognise deterioration -Doubting own ability/skills	Nurses & doctors from 1 UK hospital [23]; HCPs in 3 UK hospitals [19]; Nurses in 1 US hospital [25] Nurses in 1 Australian hospital [18]; Nurses in 1 US hospital [25]	*“Sometimes it’s overconfidence or false confidence that you think you are in control of the situation... You could spend slightly less time with a person and then go back to them and realise their condition has changed but not noticed those subtle changes because you haven’t seen them for an hour or so.”* (R6, Nurse) [23] *“Maybe questioning my decisions: Am I over-reacting here? Is this real or am I just panicking?”(Tanya)* [18] *“I think that the main thing is questioning, self-doubt.. Is the patient really sick enough to call? Can I handle this myself?”* [25]
**EWS**	**Patient variability** [9, 16, 20–22, 27, 30, 32] -Sub-populations who fall outside the normal vital sign ranges -Need for parameter adjustments	Nurses in 1 US hospital [27]; Doctors & nurses in 8 Australian hospitals [16]; Senior NCHDs & nurses in 1 Irish hospital [30]; ENs in 1 Singaporean hospital [32]; HCAs, nurses, physicians, critical care staff & managers in 2 UK hospitals [20, 21] Nurses in 1 UK hospital [22]	When asked how they would improve the current MEWS, most participants responded that they would customize the preset “normal” vital sign values to account for individual patient variances. Nurses addressed the variance by documenting that the abnormal value represented the patient’s baseline or was a desired effect of an intervention, but the system required physician notification added to nursing workload. The inability of the MEWS to tailor alarm settings and limits to accommodate patients whose vital sign measurements normally fell outside predetermined thresholds was cited by focus group participants as a major barrier to effective use of the system [27]. Participants reported that parameters were rarely reviewed or adjusted and that this was a continual problem for interns and nurses *“If parameters aren’t charted you’re expected to check the observation and inform the intern more than is necessary”* (Nurse 4) [30].

**Legend:** EN: Enrolled nurse; EWS: Early warning system; HCA: Healthcare assistant; HCP: Healthcare Professional; ICU: Intensive care unit; MET: Medical emergency team; NCHD: Non consultant hospital doctor; NEWS: National Early warning System; RRT: Rapid response team; UK: United Kingdom; US: United States

#### Governance

‘Governance’ refers to the overall organisational or institutional specific factors affecting why HCPs fail to escalate, or barriers to escalation. Fourteen papers described governance issues as factors contributing to a failure to escalate care [9, 16–21, 23–26, 29, 30, 32]. Three sub-themes including *Standardisation*, *Resources* and *Lack of accountability* were identified.

‘Standardisation’ was an issue reported in twelve studies [16–20, 24, 25, 29, 30, 32]. Standardisation included a lack of clear policies or protocols for action which was reported in four studies [19, 20, 24, 29] and this led to inaction or confusion amongst staff as to who to call or when. In addition to a lack of clear policies or protocols, ‘standardisation’ included a lack of knowledge of policies or protocols by staff, reported in six studies [16–19, 25, 29]. Where staff were not familiar with the correct protocol for escalation this was a barrier to escalation. Lack of education or training was reported in six studies by participants with no standardised, or regular training in place [17, 18, 24, 25, 30, 32].

‘Resources’ were reported as barriers in nine studies [9, 16, 19, 23, 25, 26, 29, 30, 32] whereby staffing shortages, particularly in conducting the required monitoring of patients, (eight studies), [9, 16, 19, 23, 26, 29, 30, 32] poor communication systems/protocols (three studies) [16, 19, 30] and the perceived workload of the RRT (six studies) [16, 19, 23, 25, 30, 32] were all reported as barriers to escalation: “Perceived busyness of the ICU nurses discouraged participants from RRT activation. Participants noted that responding RRT members occasionally talked about how busy they were.”

‘Lack of accountability’ and a blame culture was a reported sub-theme in three papers [20, 21, 32]. This was particularly the case in settings where health care assistants (HCAs) or equivalent staff were involved in documenting patient vital signs. HCAs believed there was often blame put on them by more senior staff when something went wrong. For example, junior staff described situations where a patient deteriorated and they informed senior staff, but the senior staff did not escalate care, and then when the patient collapsed or deteriorated the blame was put on the junior staff member [20, 21, 32]. This lack of accountability of senior staff was a barrier to these staff in raising concerns about deterioration.

#### RRT response

‘RRT Response’ refers to how the RRT responded when a call for help was made. This key theme was apparent in ten papers [9, 17–19, 24–26, 28, 29, 31]. Two sub-themes including *RRT behaviours* and *Fear* were identified.

‘RRT behaviours’ were a barrier to escalation or subsequent escalation calls when a ‘lack of professionalism’ was shown by the RRT to the staff who made the call. This was reported in eight papers [9, 17–19, 25, 28, 29, 31]. A ‘negative response’ or a total ‘lack of response’ (i.e. the RRT did not come) was also a barrier to escalation or subsequent escalation reported in eight papers [9, 17–19, 24, 25, 28, 31]. Participants reported being questioned as to whether the call to the RRT was necessary, they often reported feeling belittled or criticised and the experience of this negative response was a barrier to subsequent escalation.

Participants reported ‘fear’ was a barrier to escalation in seven papers [9, 17–19, 25, 26, 29]. ‘Fear of reprimand’ by members of the RRT for activating a call was reported by participants as well as ‘fear of looking stupid or dumb’ to colleagues, both of which were significant barriers to escalation.

#### Professional boundaries

‘Professional boundaries’ refers to the barriers to escalation that were apparent in the included studies surrounding hierarchy, power, and jurisdictional control. Ten papers described professional boundaries as core contributing factors to not escalating [17–21, 23, 25, 26, 29, 30]. Two sub-themes including *Hierarchy* and *Increased conflict* were identified.

Participants described having to negotiate hierarchical boundaries in order to escalate care in eight papers [17, 19–21, 24–26, 29]. In some instances, participants described being reprimanded by the patient’s primary ward physician for calling the RRT. The primary ward physician often felt it was “*their patient and their job to look after them*” and that the junior staff had “*gone over their head*” in calling the RRT [26, 34]. This in turn led to an increase in conflict between nurses and ward physicians. Calling for help (escalation) also led to increased conflict among other staff [17, 18, 20, 23, 25, 26, 30]. In particular, the use of the RRT was often viewed as a jurisdictional shift in responsibility for acutely ill patients by members of the RRT who felt some nurses “washed their hands” of the situation. This may contribute to the negative responses of RRT, as described above.

#### Clinical experience

‘Clinical experience’ refers to the barriers to escalation specifically related to individual staff and their level of confidence and ability to detect deterioration, which was reported in six studies [9, 18, 19, 23, 25, 29]. Two sub-themes including *Clinical over confidence* and *Lack of clinical confidence* were identified.

‘Clinical over confidence’ reported in five papers, [9, 19, 23, 25, 29] was characterised by participants being overly confident in their clinical ability. Participants expressed confidence that their clinical judgement was a better gauge of when to escalate care, irrespective of the EWS, and also that they were better placed to care for their own patient rather than the RRT.

In contrast, ‘lack of clinical confidence’, was reported in three studies [18, 25, 29]. Here it was participant’s inability to detect deterioration or doubting their own skills and ability to detect deterioration that led to a delay in escalation or to no escalation.

#### Early warning system parameters

‘EWS Parameters’ refers to the system specific barriers to escalation, which were reported in eight studies [9, 16, 20–22, 27, 30, 32]. One sub-theme, *Patient variability* was identified.

‘Patient variability’ that is the existence of specific groups of patients, for example, those with chronic obstructive pulmonary disease, was reported as a barrier. For these patients, who by default fall outside the normal range for the various vital signs, participants reported either excessive triggering of the EWS or else staff simply ignored the EWS for these patients. “The inability of the MEWS to tailor alarm settings and limits to accommodate patients whose vital sign measurements normally fell outside predetermined thresholds was cited by focus group participants as a major barrier to effective use of the system” [27]. The need for parameter adjustment was also cited within the patient variability sub-theme: participants reported that parameters were rarely reviewed or adjusted and that this was a continual problem for interns and nurses “*If parameters aren’t charted you’re expected to check the observation and inform the intern more than is necessary*” [30].

The themes of ‘governance’, ‘professional boundaries’, ‘RRT Response’, ‘Clinical Experience’, and ‘Early Warning System Parameters’ are individual but inter-related barriers to escalation of care. Each theme may be its own barrier, but when taken together they create an environment in which escalation of care may occur too late or not occur at all. For example, a lack of governance such as a lack of clear policies or protocols, or lack of knowledge of policies or protocols by all staff creates the potential for conflicts in professional boundaries. This may create a level of ‘fear’ for junior staff, particular those with less clinical confidence, who experience negative attitudes from both the RRT and primary ward physicians which contribute to a reluctance to activate the RRT in the future.

### Facilitators to escalation

Illustrative quotations from primary study participants or study authors relating to the facilitators of escalation for each key theme (*n* = 5) and sub-theme (*n* = 12) are presented in Table 3.

**Table 3:** Key themes of facilitators of escalation amongst healthcare professionals

Key Themes (Finding)	Sub-themes and references	Characteristics of studies from which sub-themes were derived: Type of participant and setting (Reference)	Illustrative quotations (*Italicised text* = primary quote from a study participant; non-italicised text = secondary quote from study authors)
**Governance**	**Accountability** [19–21, 30]	Senior resident surgeons, surgical postgraduates year 1, intensivists, and critical care outreach team members from 3 UK hospitals [19]; Year 1 interns, Senior NCHDs and nurses in 1 Irish hospital [30]; HCAs, nurses, physicians, critical care staff and managers in 2 UK hospitals [20, 21]	“*If you don’t follow the NEWS and something goes wrong then the blame rests on you and you’ve got nothing to back you up…wheras, once you call you’re protected*” [30]
	**Standardisation** -Clear policies or protocols [16, 20–22, 25, 29, 30] -Knowledge of protocols/policies [20, 21, 30]	Nurses in 1 US hospital [25]; Mainly doctors and nurses in 8 Australian hospitals [16]; Nurses in 1 UK hospital [22]; Year 1 interns, Senior NCHDs and nurses in 1 Irish hospital [30]; HCAs, nurses, physicians, critical care staff and managers in 2 UK hospitals [20, 21]	*“I will continue to use it as I’m currently using it unless the protocol changes as it’s a requirement of my job and part of the hospital’s policy (Nurse 8)”* [30]. Both the escalation protocol and the CCOT at Westward promoted uniformity and standardisation with regards to response to the acutely ill patient [20]. *“As soon as we get a high score we’re supposed to go straight to the staff nurse and inform them that this patient’s observations have been outside normal. And then the staff nurse will inform the doctor and say, ‘this patient’s blood pressure is below normal, is x, y, z, so if you could come and review this patient.”* [21]
	**Resources** -Sufficient staffing/reduced workload [19, 23, 25, 30] -Good communication [19, 20, 22, 25, 27, 30]	Nurses in 1 US hospital; (25) Nurses and doctors in 1 UK hospital [23]; HCPs from 3 UK hospitals [19]; HCPs from 1 Irish hospital [30]; HCPs from 2 UK hospitals [20]; Nurses from 1 UK hospital [22]; Nurses from 1 US hospital [27].	*“There is now a single resident who covers the ward for the week and twice daily attending ward rounds. I think this has made things better for juniors because they have a single point of contact who is not going to be off site or in theatre” (Surgeon)* [19] The team used SBAR, the communication technique approved by the facility…. Standardised language helped participants provide information quickly and accurately [25].
**RRT Response**	**RRT Behaviours** - Professionalism/Positive responses [9, 19, 25, 29, 31] -Decision-makers/Doers [19, 25, 31] -Collaborative [23, 28, 31]	Nurses in 1 US hospital [25]; HCPs in 3 UK hospitals [19]; Nurses in 1 Norwegian hospital [31]; Nurses and doctors in 1 UK hospital [23]; Nurses in 1 US hospital [28]; HCPs in 1 US hospital [26]; Doctors and nurses in 4 Australian hospitals [17]; HCPs in 2 UK hospitals [20] Nurses in 1 Norwegian hospital [31]; Nurses and doctors in 1 UK hospital [23]; Nurses in 1 US hospital [28]	The approachable style and non-critical attitude of the MICN and their prompt responses in giving advice over the phone or reviewing the situation in person were recurrent comments throughout the interviews [31]. *“ICU nurses’ expertise is reassuring. They evaluate the situation. They figure out what is going on and decide what to do”* [25]. “*The MICN did not ‘take over’ the situation, he only confirmed and asked for collaboration by using skills in communication and support and gave us treatment suggestions. We learned and listened; hopefully I can use this knowledge in other situations too*” [31].
	**Expertise** (Skilled) [9, 23, 25, 26]	Nurses and doctors in 1 UK hospital [23]; Nurses in 1 US hospital [25]; HCPs in 1 US hospital [26]	Nurses had a sense of security and of empowerment generated by knowing skilled backup was available immediately through a single phone call [26].
	**Additional Support** [17, 20, 23, 25, 26, 28, 29, 31]	Nurses and doctors in 1 UK hospital [23]; Doctors and nurses in 4 Australian hospitals [17]; Nurses in 1 Norwegian hospital [31]; HCPs in 2 UK hospitals [20]; Nurses in 1 US hospital [25]; HCPs in 1 US hospital [26]; Nurses in 1 US hospital [28]	‘.. .*an extra pair of eyes and ears for patients who are at risk of deteriorating or are in the process of deteriorating; and really able to bring critical care experience to a ward environment, to support the nurses and doctors on the ward to care for deteriorating patients on the ward. It’s a very supportive role, bringing that extra degree of knowledge and skills that we may not have on the ward to care for the patient*.’ (R7, Nurse) [23]
**Professional Boundaries**	**Licence to escalate** (Autonomy) [16–20, 23, 26, 29, 30]	Nurses and doctors in 1 UK hospital [23]; Mainly doctors and nurses in 8 Australian hospitals [16]; Doctors and nurses in 4 Australian hospitals [17]; Nurses in 1 Australian hospital [18]; Year 1 interns, Senior NCHDs and nurses in 1 Irish hospital [30]; HCPs in 3 UK hospitals [19]; HCPs in 2 UK hospitals [20]; HCPs in 1 US hospital [26]	Across both sites the score provided staff with the licence to escalate care across hierarchical and occupational boundaries [20]. *“The nurses actually have something they can do about it* versus *just kind of watching the patient flounder (hospitalist)”* [26]
	**Bridge Across Boundaries** (Facilitates cross-profession communication and teamwork, workaround) [17, 19, 20, 26]	Doctors and nurses in 4 Australian hospitals [17]; HCPs in 3 UK hospitals [19]; HCPs in 2 UK hospitals [20]; HCPs in 1 US hospital [26]	*“We used to actually use them as a way of getting round a resident or whoever really wasn’t doing what you know you needed for your patient (Junior nursing, site 2)”* [17] The EWS helped with escalation of care across boundaries: *“The score is useful….if you’re handing over the phone in the middle of the night to someone you’ve never met before….they don’t know your judgement and your experience, so it’s kind of a physical....this is quite clear” (Nurse, 5, Westward)* [20]
**Clinical Experience**	**Clinical Confidence** [16, 22, 25, 27, 29] **-**To recognise deterioration -Confidence in own ability and skills	Nurses in 1 US hospital [25]; Nurses and doctors in 8 Australian hospitals [16]; Nurses in 1 UK hospital [22]; Nurses in 1 US hospital [27]	*“I’d like to think that it hasn’t made any difference to me being able to detect my patient deteriorating (FG I1)” and “I went to nursing school for three years - I know when it is time to ring the doctor” (FG A4)* [16] *“I never hesitate to call an RRT because I’m afraid I’ll be criticized or made to feel like I couldn’t handle a situation.”* [27]
	**Empowerment/validation** [16, 17, 19, 20, 22, 26, 28, 30]	Mainly doctors and nurses in 8 Australian hospitals [16]; Doctors and nurses in 4 Australian hospitals [17]; Year 1 interns, Senior NCHDs and nurses in 1 Irish hospital [30]; HCPs in 3 UK hospitals [19]; HCPs in 2 UK hospitals [20]; Nurses in 1 UK hospital [22]; HCPs in 1 US hospital [26]; Nurses in 1 US hospital [28]	Availability of the RRT empowered nurses who were able to obtain additional help without having to request permission. “*I don’t usually hesitate to call. I notify the team of any changes, and if I feel like I need additional nursing support or if I need respiratory support right that minute, I will call an RRT”* [26].
	**Clinical judgement** [9, 16, 17, 19, 25, 29, 30]	Mainly doctors and nurses in 8 Australian hospitals [16]; Doctors and nurses in 4 Australian hospitals [17]; Year 1 interns, Senior NCHDs and nurses in 1 Irish hospital [30]; HCPs in 3 UK hospitals [19]; Nurses in 1 US hospital [25] Nurses in 1 Australian hospital [18]; Nurses and doctors in 1 UK hospital [9]	Participants referred to the importance of using clinical judgement in tandem with the RRT criteria to guide their assessment and decision-making processes when deliberating whether or not to activate the RRT [17]. “*She just had this sweaty clammy look and just going from previous experience again, it was like there is something really not right here.”* (R1, Nurse) [23]
**EWS Parameters**	**Triage mechanism/Tool for detecting deterioration** [9, 18, 20, 22, 25–27, 30]	Nurses in 1 US hospital [27]; Nurses in 1 Australian hospital [18]; Year 1 interns, Senior NCHDs and nurses in 1 Irish hospital [30]; HCPs in 2 UK hospitals [20]; Nurses in 1 UK hospital [22]; HCPs in 1 US hospital [26]; Nurses in 1 US hospital [25]	Doctors described using the system to gauge the severity of a patient’s condition for triaging: “*When I’m contacted to review a patient, I use ‘NEWS’ to prioritise the urgency in which they need to be reviewed* (NCHD 2)” [30] All staff valued the training they had received and reported that the T&T helped identify patient deterioration earlier: *“We now use it on every single patient that we have on the ward and obviously they all get a score at the end of it, so I think it just rings more alarm bells if you like if a patient is unwell or is deteriorating, whereas just recording a patient’s observations, you know, you might miss something* [15] *It does highlight patients that are actually deteriorating quicker than you would”* [22].

**Legend: OT:** Critical care outreach team; **EWS:** Early warning system; **HCA:** Healthcare assistant; **HCP**: Healthcare Professional; **MICN:** Mobile intensive care network; **NCHD**: Non consultant hospital doctor; **NEWS**: National Early warning System; **RRT/S:** Rapid response team/system; **SBAR**: Situation, Background, Assessment, Response; **UK:** United Kingdom; **US:** United States.

#### Governance

‘Governance’ was a key theme within ten papers [16, 19–23, 25, 27, 29, 30]. Three sub-themes of ‘*Accountability*’, ‘*Standardisation*’ and ‘*Resources*’ as facilitators of escalation among the study participants were identified.

Accountability was a motivating factor in four studies, whereby staff activated the RRT in order to ‘cover their own backs’ in case something went wrong [19–21, 30]. In this respect, the RRT was viewed as a safety net by the nurses and they valued the extra support it provided.

In addition, ‘standardisation’ was reported in seven studies, where clear policies or protocols for action [16, 20–22, 25, 29, 30] and participant knowledge of these policies or protocols for escalation [20, 21, 30] was a key facilitator of escalation. A clear outline of when to call and who to call, that was communicated to and understood by all staff members, was a facilitator of escalation.

Resources (that is sufficient staffing levels and good communication such as use of handover tools) was a key facilitator of escalation in seven studies, [19, 20, 22, 23, 25, 27, 30] as exemplified here: “*There is now a single resident who covers the ward for the week and twice daily attending ward rounds. I think this has made things better for juniors because they have a single point of contact who is not going to be off site or in theatre*” [19].

#### RRT response

The behaviours of RRTs were reported as facilitators of escalation within this key theme in ten studies [9, 17, 19, 20, 23, 25, 26, 28, 29, 31]. Three sub-themes of ‘*RRT behaviours*’ (including professionalism, decision-makers and collaborative), ‘*Expertise*’ and ‘*Additional support*’ were identified.

In terms of RRT behaviours, where there was a ‘professional response’ or a ‘positive response’ from the RRT, this encouraged staff to escalate in subsequent events [9, 19, 25, 29, 31]. The RRT were seen as ‘decision-makers’ and ‘doers’ in emergency situations and these were both facilitators of escalation [19, 25, 31]. The RRT were viewed as collaborative but also of facilitating collaboration between staff, and this was another facilitator of escalation within three studies [23, 28, 31].

In addition to how the RRT behaved, they were also described as being ‘experts’ [9, 23, 25, 26] with specific specialised skills and expertise necessary when a patient deteriorated.

They were also seen as providing ‘additional support’ [17, 20, 23, 25, 26, 28, 29, 31] in emergency situations and this was a source of comfort reported by participants.

#### Professional boundaries

Professional boundaries as a key theme was included in nine studies [16–20, 23, 26, 29, 30]. This included the sub-themes of a ‘*Licence to escalate’* and a ‘*Bridge across boundaries’*.

Licence to escalate was where the staff perceived the EWS as tool to enable escalation across hierarchical and occupational boundaries and was apparent in nine studies, [16–20, 23, 26, 29, 30] as exemplified from the following extracts: “Across both sites the score provided staff with the licence to escalate care across hierarchical and occupational boundaries” [20]. *“The nurses actually have something they can do about it versus just kind of watching the patient flounder”* [26]. The EWS was used as a tool by nurses to establish a legitimate reason for escalating care to more senior staff without having to seek permission. This licence created a ‘Bridge across boundaries’. This refers to the view that the EWS facilitates cross-profession communication and teamwork and is a workaround and means of getting something done, i.e. getting a patient seen to, and was referenced in four studies [17, 19, 20, 26]. *“We used to actually use them as a way of getting round a resident or whoever really wasn’t doing what you know you needed for your patient”* [17].

#### Clinical experience

Clinical experience was a key theme within 12 studies and included sub-themes of ‘*Clinical confidence*’ (to recognise deterioration, confidence in own ability and skills), ‘*Empowerment/validation’* and ‘*Clinical judgement’* [9, 16–20, 22, 23, 25–30].

Where a staff member had clinical confidence in their own skills and ability and were able to recognise deterioration, this was a facilitator of escalation. Staff were confident enough to activate the RRT [16, 22, 25, 27, 29].

Staff also felt ‘empowered’ by the EWS and the EWS ‘validated’ their reasons for escalation and calling for help from the RRT and seniors [16, 17, 19, 20, 22, 26, 28, 30].

‘Clinical judgement’ was a facilitator of escalation in seven studies where staff referred to the importance of using clinical judgement when a patient deteriorates and not relying on a score or system alone [9, 16, 17, 19, 25, 29, 30].

#### Early warning system parameters

The fifth key theme of EWS Parameters included the subtheme of ‘Triage mechanism’ and a ‘Tool for detecting deterioration’. [9, 18, 20, 22, 25–27, 30].

Staff described using the EWS as a mechanism for triage, to get a patient a higher level of care and to ensure patient safety. In addition, the EWS was seen as a valuable tool for picking up patient deterioration by staff and optimising patient outcomes. Doctors described using the system to gauge the severity of a patient’s condition for triaging: “*When I’m contacted to review a patient, I use ‘NEWS’ to prioritise the urgency in which they need to be reviewed* [30].

Just as the themes of ‘governance’, ‘professional boundaries’, ‘RRT Response’, ‘Clinical Experience’, and ‘Early Warning System Parameters’ were inter-related in the generation of barriers to escalation of care, the themes are inter-related in creating facilitators to the escalation of care in addition. For example, clear governance in terms of policies or protocols, and knowledge of policies or protocols by all staff decreases the potential for conflicts in professional boundaries and increases role clarity. This in turn may create a more collaborative team-based approach that provides reassurance and confidence as opposed to engendering a level of ‘fear’ in junior staff, particularly those with less clinical confidence. All of which combines to create a climate within which activation of the RRT is more likely to happen.

### Quality appraisal

The CASP tool was used for quality appraisal (see Additional file 3). All 18 studies reported a clear statement of the aims. All 18 studies were judged to have used an appropriate qualitative methodology [e.g. focus groups or interviews], and were judged to have employed appropriate data collection methods (e.g. interviews or focus groups or observation techniques or document review). All studies had a clear statement of the findings and the research was deemed valuable. Seven out the 18 studies were judged to have a research design appropriate to the study aims, [17, 18, 21, 23, 29, 30, 32] while in 11 of 18 studies there was insufficient information on the rationale for the chosen qualitative methodology [9, 16, 19, 20, 22, 24–28, 31]. Thirteen out of the 18 studies were judged to have a recruitment strategy appropriate to the study aims (e.g. convenience sampling or purposeful sampling), [9, 16–23, 25, 26, 29, 31] and in four studies there was insufficient information provided [24, 27, 28, 32]. In one study the recruitment strategy was deemed inappropriate (the study authors used ‘their judgement’ and snowball techniques) [30]. Six out 18 studies considered the researcher and participant relationship within the study, [9, 18, 20, 24, 27, 28] while 11 out the 18 studies did not consider the researcher-participant relationship and the potential for bias this may introduce [16, 17, 19, 21–23, 26, 29–32]. In one study insufficient information was provided [25]. Seventeen out of the 18 studies reported having ethical approval while in one study it was unclear [27]. Fifteen out of 18 studies were judged to have rigorous data analysis (e.g. inductively and deductively coded, content analysis), [9, 17, 18, 20–24, 26–30, 32] while in two studies there was insufficient detail provided within in the study (the authors mentioned triangulation but provided no other details and in the second study no coding framework was provided) [19, 25]. In one study, the analysis was deemed insufficient as there were missing observations which were not reported [31].

### Certainty of the evidence

Confidence in the review findings was assessed using GRADE-CERQual, [13] Overall the certainty of the evidence ranged from moderate to high (see Additional file 4) and there was strong consistency in the findings across studies. Confidence in review findings were downgraded primarily due to methodological limitations.

## Discussion

This qualitative evidence synthesis identified 18 studies from various countries, all conducted in hospital settings and including nurses only (ten studies), nurses and doctors only (three studies) or a mix of HCPs and staff (including administrators, management, allied health professionals, etc.), (six studies). The studies reported participant’s views, beliefs and opinions on various EWSs or rapid response systems using mainly face-to-face interviews or focus group techniques.

A comprehensive thematic analysis resulted in the generation of five key themes as barriers and facilitators to escalation: *Governance, RRT Response, Professional Boundaries, Clinical Experience* and *Early Warning System Parameters*. The theme of governance and the sub-themes - standardisation, accountability and resources – was seen as both a facilitator and a barrier to escalation. Clear policies and protocols for escalation and HCP knowledge of these policies and protocols was a key facilitator of escalation. Clear organisational policy as to who to call and when and communication of this information to HCPs was also reported to be a facilitator of escalation. The converse was also found where lack of such policies and/or lack of HCP knowledge of these policies was a barrier to escalation. Nursing staff used the escalation policies ‘to cover their backs’ and valued the back-up provided by the RRT when they escalated care. Sufficient staffing levels and use of communication tools facilitated escalation of care while staff shortages and poor communication processes were impediments to escalation. These findings emphasise the importance of ‘closed loop governance’ at senior clinical and organisational levels.

The RRT was seen as a facilitator to escalation when RRT member behaviours were positive and professional. The RRT were considered by ward staff to be expert clinical decision-makers in emergency situations who facilitated collaboration amongst staff. Negative behaviours by RRT members and fear of a negative response or of looking stupid were barriers to escalation.

Professional boundaries was both a facilitator and a barrier to escalation. Acknowledging the hierarchical nature of healthcare nursing staff perceived the escalation protocol as a license to escalate care across hierarchical and occupational boundaries. In this way the EWS facilitated cross-professional communication and teamwork and was a means of getting something done. On the other hand, ward physicians often perceived that use of the escalation protocol was going above their head in relation to their patients. In some instances members of the RRT felt that escalation of care by nursing staff was shifting the responsibility for an acutely ill patient from ward staff to the RRT.

Clinical experience was paradoxically both a facilitator and a barrier to escalation. HCPs’ confidence in their ability to recognise clinical deterioration influenced their decision to escalate while over-confidence in their clinical ability resulted in staff relying on their clinical judgement and deciding not to escalate despite an elevated EWS score.

EWS parameters developed as the fifth theme specifically around patient variability. EWSs are designed to maximise discrimination between patients at risk of adverse outcomes (death, cardiac arrest or unplanned admission to ICU) and those not at risk of these outcomes. The use of EWSs in specific patient cohorts, in particular those with chronic respiratory conditions whose baselines often fall outside of EWS physiological parameter ranges, can result in over-triggering of the system and more frequent alerts than necessary. This can cause alarm fatigue and inefficient use of the nursing and medical workforce resource. This was seen as major barrier to effective use of the system. It was also a significant patient safety issue as it led to the adjustment of EWS parameters - effectively removing a patient from an EWS – and these parameter adjustments were rarely reviewed.

### Comparison with the literature

A previous systematic review by Chua et al. synthesized evidence on the factors influencing rapid response team (RRT) activation by junior physicians and ward nurses using the Systems Engineering Initiative for Patient Safety (SEIPS) model of work system and patient safety as a conceptual framework and found that the elements of person, tools and technologies, tasks, and organization were associated with RRT activation [35]. Similar to our qualitative evidence synthesis review findings, the authors too found that ward nurses’ adherence to the traditional model of escalation of care was associated with their fear of criticism for ‘incorrect’ activations. This fear of criticism is in turn linked to insufficient clinical experience (person-related), inadequacy in the activation criteria (tools and technological-related), and dismissive responses from RRT members (task-related), which often leads to ward nurses seeking affirmation that they had acted correctly by activating the RRT*.* Again, similar to our findings, experienced nurses were found to be more capable and confident in recognising the need for RRT intervention. Similar to the ‘professional boundaries’ theme and ‘hierarchy’ sub theme we reported, adherence to the traditional model of calling attending physicians first was the biggest barrier for junior physicians in this previous review. Their findings suggest that this barrier could be attributed to their perception of threatened deskilling due to the presence of the RRT*.* Resistance from the medical profession towards the acceptance of the RRT due to perceived disruptive effects on junior physicians’ education and clinical autonomy can be linked to physicians who lay claims to their expertise and jurisdictions over patient management, again a similar finding to our review where clinician’s claimed ownership over ‘their patients’.

A further scoping review by Wood et al., [36] identified three themes from the qualitative synthesis namely, inconsistent activation of the rapid response team; barriers to following EWS algorithms; and overreliance on scores. The authors report that at times there was a reluctance amongst HCPs to activate the RRT when they were concerned about a patient or if they met the actual criteria on the EWS track and trigger chart. Several factors influenced whether or not HCPs activated the RRT including nurses’ self-confidence (clinical confidence in our review), past experience with the RRT (RRT response in our review) and fear of retribution or criticism from the RRT if the call was made and the patient wasn’t critically unwell (RRT behaviour and fear sub themes in our review). Similar to our review findings, the likelihood of nurses activating the RRT is acutely intertwined with their confidence and previous experiences with the RRT with more experienced nurses increasingly likely to use clinical judgment and enable the effective use of EWSs whilst poor skill mix levels (less clinical experience) constrained the optimal use of EWSs.

Furthermore, a previous review of educational interventions aimed at nurses and focusing on the use of EWSs and the management of deteriorating patients, reported that they increase awareness, knowledge and management of deteriorating patients [37]. This previous review, in line with our review findings (Theme: Governance, subtheme Lack of standardised education and training) found that nurses frequently raised concerns about their ability to adhere to the algorithms incorporated into EWS track and trigger charts that aim to guide them through when to escalate in the event of patient deterioration. The nurses expressed difficulty in getting medical officers to review patients with high scores due to their lack of familiarity and understanding of the charts and algorithms. Lack of resources was also cited with nurses indicating they cannot follow the algorithms due to high workloads or lack of equipment and could indicate health services do not have adequate resources or systems in place that ensure EWS algorithms can be followed. It appears that there are several factors at play that prevent HCPs from using the EWS appropriately as our review and the existing literature demonstrates.

### Implications for policy and practice

It is clear that the findings of our qualitative evidence synthesis add to the existing evidence and demonstrate that the barriers and facilitators to escalation are intertwined and multifactorial. It is obvious that resources are a concern, and that lack of clear, standardised protocols or lack of knowledge of such protocols is something that needs to be addressed by our policy makers and in practice on the frontline. Standardised education across all disciplines and not solely nursing staff needs to be prioritized. What was most worrying from the review findings, is the socio-cultural barriers including the hierarchical barriers to escalation of care, as well as fear of criticism and negative behaviors of the RRT responders. This needs to be addressed by hospital management and clinical team leaders and through the continuous training of HCPs across all grades and levels.

Education and training is required to encourage clinicians and teams to respond responsibly, to establish and follow clear protocols, and to provide continuous support to advocate for the escalation of care and activate the RRT where required. The findings of this qualitative evidence synthesis have been used to update the Irish National Clinical Guideline No.1 Irish National Early Warning System (INEWS V2) and has resulted in recommendations been made for standardised education for all HCPs as well as the inclusion of the following text to the INEWS Escalation and Response protocol “*if response does not occur as per protocol the Clinical Nurse Manager/Nurse In Charge should escalate directly to the Consultant*”, enabling the nurse to escalate above the ward doctor if patient care is a concern. The recommendation in the revised guideline for a new three-tiered response service clarifies the process of escalation for HCPs by delineating bedside, urgent and emergency escalation levels. In relation to the findings on EWS parameters a new modified escalation and response protocol was co-designed with an expert consultant advisory group to manage patients whose baseline observations fall outside of EWS physiological parameter ranges. To assist in the recognition of deterioration and thereby addressing the finding ‘lack of clinical experience’ and ‘doubt by staff of their ability to recognise deterioration’ an additional step was added to the Irish EWS - ‘anticipation’ - which provided staff with a series of prompts to enable them to consider the broader clinical context when assessing patients. Thus INEWS becomes anticipation, recognition, escalation, response and governance.

### Strengths and limitations of this systematic review

The strengths of this systematic review include the thorough searching of the literature (more than 10 electronic databases as well as grey literature resources and over 30 websites) using an extensive search strategy with the added benefit of a librarian to assist with the search and provide expertise. In addition, data extraction and quality appraisal as well as the highly recommended GRADE CERQual approach were conducted by two reviewers independently using standardised data extraction forms and quality appraisal tools to ensure rigour and reduce bias. The limitations of the review include firstly that the primary search was conducted up to February 2018. However, due to the large number of studies included from a variety of contexts and the moderate to high confidence in the findings, the authors concluded that the addition of new studies was unlikely to substantially change the findings. In addition, date (January 2011 to February 2018) and language restrictions (English language only) were applied. However, given that meaning may be lost in translation and that there was a breadth of countries and contexts covered by the  included studies, the exclusion of non-English studies may not a represent a large bias [38]. Despite the broad search strategy, some studies may not have been retrieved due to difficulties identifying qualitative literature [39].

## Conclusion

Delays in providing care to deteriorating hospitalised patients increases the likelihood of serious adverse events including unanticipated cardiopulmonary arrest, unplanned admissions/readmission to ICU and death. Emergency response systems evolved internationally to assist HCPs in recognising and managing the acutely unwell hospitalised patient. EWSs incorporate escalation and response protocols, which enable bedside HCPs to rapidly escalate care of the deteriorating patient for more senior clinical review. This qualitative evidence synthesis focussed on why HCPs fail to escalate as per the escalation protocol, and aimed to identify the barriers and facilitators to escalation from a thematic analysis of the literature. The findings of this qualitative evidence synthesis provide insight into the real world experience of HCPs when using EWSs. This in turn has the potential to inform policy-makers and HCPs as well as hospital management about emergency response system - related issues in practice and the need to incorporate changes as a result of these findings to improve patient safety and quality of care.

## Supplementary Information

**Additional file 1.**

**Additional file 2.**

**Additional file 3.**

**Additional file 4.**

## Data Availability

All data generated or analyzed during this study are included in this published article [and its supplementary information files].

## Abbreviations

**CASP**: Critical Appraisal Skills Programme
**CERQual**: Confidence in the Evidence from Reviews of Qualitative Research
**ENTREQ**: Enhancing Transparency in reporting the synthesis of qualitative research
**EWS**: Early warning system/score
**HCA**: Health care assistant
**HCP**: Health care professional
**HDI**: Human Development Index
**HIQA**: Health Information and Quality Authority
**HRB-CICER**: Health Research Board Ireland Collaboration in Ireland for Clinical Effectiveness Reviews
**ICU**: Intensive Care Unit
**INEWS**: Irish National Early Warning System
**MET**: Medical Emergency Team
**MEWS**: Modified Early Warning Score/System
**NCHD**: National Clinical House Doctor
**NEWS**: National Early Warning System/Score
**RRT**: Rapid Response Team
**UK**: United Kingdom
**USA**: United States of America
