# Supplementary material for: Why do healthcare professionals fail to escalate as per the early warning system (EWS) protocol? A qualitative evidence synthesis of the barriers and facilitators of escalation
Source: BMC Emerg Med. 2021 Jan 28;21:15. doi: 10.1186/s12873-021-00403-9 (PMC7842002; doi:10.1186/s12873-021-00403-9)
Supplement: Supplementary file 1 — Additional file 1. [file 12873_2021_403_MOESM1_ESM.docx]

**Electronic databases searched (From January 2011-February 19^th^ 2018)**

| 1. Academic Search Complete 2. Cumulative Index to Nursing and Allied Health Literature (CINAHL) 3. Applied Social Sciences Index and Abstracts (ASSIA) 4. Medical Literature Analysis and Retrieval System Online (MEDLINE) 5. PsycARTICLES 6. PsycINFO 7. Psychology and Behavioral Sciences Collection 8. SocINDEX 9. Exerptamedica Database (EMBASE) 10. Health Management Information Consortium (HMIC) 11. The Cochrane Library ([www.cochranelibrary.com](http://www.cochranelibrary.com)) |
| --- |

**Grey Literature resources searched (From January 2011-February 19^th^ 2018)**

| **Databases:**   1. OpenGrey System for Information on Grey Literature in Europe (<http://www.opengrey.eu/>) 2. Open University Dedicated Grey Literature site (<http://www.open.ac.uk/library/>) 3. Education Resources Information Center (ERIC) database   (<https://eric.ed.gov/>)   1. GrayLit Network (via Science.Gov as it was discontinued in 2007 and archived in Science.Gov)   (<https://www.science.gov/>)   1. Networked Digital Library of Theses.   (<http://www.ndltd.org/>)  **Websites:**   1. Agency for Healthcare Research and Quality   (<https://www.ahrq.gov/>)   1. Andalusian Agency for Health Technology Assessment (AETSA)   (<http://www.inahta.org/>)   1. Association of Anaesthetists of Great Britain and Ireland   (<https://www.aagbi.org/>)   1. Australian National Health and Medical Research Council Clinical Practice Guidelines   (<https://www.nhmrc.gov.au/>)   1. Belgian Health Care Knowledge Centre   (<https://kce.fgov.be/en>)   1. Canadian Medical Association InfoBase of Clinical Practice Guidelines   (<https://www.cma.ca/En/Pages/clinical-practice-guidelines.aspx>)   1. eGuidelines (UK)   (<https://www.guidelines.co.uk/>)   1. Danish Health Authority/Danish Secretariat for Clinical Guidelines   (<https://www.sst.dk/en/national-clinical-guidelines>)   1. European Society of Intensive Care Medicine   (<https://www.esicm.org/>)   1. Finnish Medical Society Duodecim   (<https://www.duodecim.fi/english/>)   1. Geneva Foundation for Medical Education and Research   (<https://www.gfmer.ch/>)   1. Guidelines International Network (GIN)   (<http://www.g-i-n.net/>)   1. German Institute of Medical Documentation and Information   (<https://www.dimdi.de/static/en/index.html>)   1. Haute Autorité de santé   (<https://www.has-sante.fr/portail/jcms/r_1455081/Home-page>)   1. Institute for Healthcare Improvement (USA)   (<http://www.ihi.org/>)   1. Intensive Care Society   (<https://www.ics.ac.uk/>)   1. Intensive Care Society of Ireland   (<http://www.intensivecare.ie/>)   1. Intensive Care National Audit & Research Centre   (<https://www.icnarc.org/>)   1. Japan Council for Quality Health Care   (<https://jcqhc.or.jp/en/>)   1. National Institute for Health and Clinical Excellence (NICE)   (<https://www.nice.org.uk/>)   1. National Library for Health (NLH) Guidelines Finder/National Library for Health (NLH) Protocols and Care Pathways database (archived 2008)   (<http://webarchive.nationalarchives.gov.uk/20081113053157/https://www.library.nhs.uk/GuidelinesFinder/AboutUs.aspx>)   1. National Guideline Clearinghouse (USA)   (<https://www.guideline.gov/>)   1. NCEC (National Clinical Effectiveness Committee, Ireland)   (<http://health.gov.ie/national-patient-safety-office/ncec/national-clinical-guidelines/>)   1. New Zealand Guidelines Group   (<https://www.health.govt.nz/>)   1. NHS Evidence database (UK)   (<https://www.evidence.nhs.uk/>)   1. NHS Institute for Innovation and Improvement (ceased in 2013)   (<https://www.gov.uk/government/organisations/nhs-institute-for-innovation-and-improvement>)   1. Royal College of Physicians   (<https://www.rcplondon.ac.uk/>)   1. Royal College of Surgeons   (<https://www.rcseng.ac.uk/>)   1. The Royal College of Anaesthetists   (<https://www.rcoa.ac.uk/>)   1. Royal College of Nursing   (<https://www.rcn.org.uk/>)   1. Scottish Intensive Care Society   (<https://www.scottishintensivecare.org.uk/>)   1. Singapore Ministry of Health   (<https://www.moh.gov.sg/content/moh_web/home.html>)   1. Socialstyrelsen (Health and Medical Care and Social Services, Sweden)   (<http://www.socialstyrelsen.se>)   1. Society of Critical Care Medicine (USA)   ([http://www.sccm.org/](http://www.sccm.org/Pages/default.aspx))   1. TRIP Database   (<https://www.tripdatabase.com/>)   1. World Health Organization   (<http://www.who.int/en/>). |
| --- |
