# Supplementary material for: Why do healthcare professionals fail to escalate as per the early warning system (EWS) protocol? A qualitative evidence synthesis of the barriers and facilitators of escalation
Source: BMC Emerg Med. 2021 Jan 28;21:15. doi: 10.1186/s12873-021-00403-9 (PMC7842002; doi:10.1186/s12873-021-00403-9)
Supplement: Supplementary file 2 — Additional file 2. [file 12873_2021_403_MOESM2_ESM.docx]

**Search terms used in the systematic review to answer: Why do health care professionals fail to escalate as per the NEWS protocol?**

**Early Warning System Specific Search Terms:**

| **A**  *“Detection of deterioration” OR “clinical deterioration” OR “identification of deterioration” OR “physiological scoring system” OR “risk assessment report” OR “emergency response system” OR “early warning” OR “warning system” OR “warning scor*” OR “failure to rescue” OR “vital sign” OR “electronic system” OR “tablet” OR “iPad” OR “escalation protocol” OR “communication” OR “response” OR “VIEWS” OR “NEWS” OR “medical emergency team” OR “rapid response team” OR “rapid response system” OR “emergency response system” OR “emergency response team”.* |
| --- |

**AND**

**Qualitative Outcomes Specific Search Terms:**

| **B**  *“Failure to escalate” OR “fail to escalate” OR “non-adherence to EWS escalation protocol” OR “qualitative” OR “ethnography” OR “phenomenology” OR “grounded theory” OR “mixed methods” OR “study design” OR “interview” OR “attitudes” OR “themes”.* |
| --- |

**AND**

**Qualitative study Design Specific Search Terms:**

| **C**  “*Qualitative” OR “ethnography” OR “phenomenology” OR “grounded theory” OR “mixed methods” OR “study design” OR “interview” OR “attitudes” OR “themes”* |
| --- |

*Combined according to the principles of Boolean Logic: A AND B AND C*
