# Supplementary material for: Why do healthcare professionals fail to escalate as per the early warning system (EWS) protocol? A qualitative evidence synthesis of the barriers and facilitators of escalation
Source: BMC Emerg Med. 2021 Jan 28;21:15. doi: 10.1186/s12873-021-00403-9 (PMC7842002; doi:10.1186/s12873-021-00403-9)
Supplement: Supplementary file 3 — Additional file 3. [file 12873_2021_403_MOESM3_ESM.docx]

| **Study** | **CASP Question** | | | | | | | | | |
| --- | --- | --- | --- | --- | --- | --- | --- | --- | --- | --- |
|  | **Clear statement of the aims?** | **Qualitative methodology appropriate?** | **Research design appropriate to study aims?** | **Recruitment strategy appropriate to study aims?** | **Data collection appropriate?** | **Researcher & participant relationship considered?** | **Ethical issues considered?** | **Rigorous data analysis?** | **Clear statement of findings?** | **Is the research valuable?** |
| Astroth (2013)^(202)^ | Yes | Yes | Can’t tell | Yes | Yes | Can’t tell | Yes | Can’t tell | Yes | Yes |
| Benin (2012)^(203)^ | Yes | Yes | Can’t tell | Yes | Yes | No | Yes | Yes | Yes | Yes |
| Braaten (2015)^(205)^ | Yes | Yes | Yes | Yes | Yes | No | Yes | Yes | Yes | Yes |
| Chua (2013)^(209)^ | Yes | Yes | Yes | Can’t tell | Yes | No | Yes | Yes | Yes | Yes |
| Cherry (2015)^(201)^ | Yes | Yes | Can’t tell | Can’t tell | Yes | Yes | Yes | Yes | Yes | Yes |
| Elliott (2015)^(195)^ | Yes | Yes | Can’t tell | Yes | Yes | No | Yes | Yes | Yes | Yes |
| Johnston (2014)^(198)^ | Yes | Yes | Can’t tell | Yes | Yes | No | Yes | Can’t tell | Yes | Yes |
| Kitto (2015)^(196)^ | Yes | Yes | Yes | Yes | Yes | No | Yes | Yes | Yes | Yes |
| Lydon (2016)^(206)^ | Yes | Yes | Yes | No | Yes | No | Yes | Yes | Yes | Yes |
| Mackintosh (2012)^(199)^ | Yes | Yes | Can’t tell | Yes | Yes | Yes | Yes | Yes | Yes | Yes |
| Mackintosh (2014)^(200)^ | Yes | Yes | Yes | Yes | Yes | No | Yes | Yes | Yes | Yes |
| Massey (2014)^(197)^ | Yes | Yes | Yes | Yes | Yes | Yes | Yes | Yes | Yes | Yes |
| McDonnell (2013)^(184)^ | Yes | Yes | Can’t tell | Yes | Yes | No | Yes | Yes | Yes | Yes |
| Pattison (2012)^(101)^ | Yes | Yes | Yes | Yes | Yes | No | Yes | Yes | Yes | Yes |
| Petersen (2017)^(208)^ | Yes | Yes | Can’t tell | Yes | Yes | Yes | Yes | Yes | Yes | Yes |
| Stafseth (2016)^(207)^ | Yes | Yes | Can’t tell | Yes | Yes | No | Yes | No | Yes | Yes |
| Stewart (2014)^(60)^ | Yes | Yes | Can’t tell | Can’t tell | Yes | Yes | Can’t tell | Yes | Yes | Yes |
| Williams (2011)^(204)^ | Yes | Yes | Can’t tell | Can’t tell | Yes | Yes | Yes | Yes | Yes | Yes |

**Why do healthcare professionals fail to escalate as per the NEWS protocol? Quality Appraisal of included studies**
