# Supplementary material for: Why do healthcare professionals fail to escalate as per the early warning system (EWS) protocol? A qualitative evidence synthesis of the barriers and facilitators of escalation
Source: BMC Emerg Med. 2021 Jan 28;21:15. doi: 10.1186/s12873-021-00403-9 (PMC7842002; doi:10.1186/s12873-021-00403-9)
Supplement: Supplementary file 4 — Additional file 4. [file 12873_2021_403_MOESM4_ESM.docx]

**GRADE CERQual Assessing the Certainty of the Evidence**

For the 18 included qualitative studies, the GRADE-CERQual (Confidence in the Evidence from Reviews of Qualitative research) approach was used to summarise confidence in the evidence.^(37)^ Four components contribute to an assessment of confidence in the evidence for each key finding (in this review, the key findings are the five key themes generated from the thematic analysis): methodological limitations, relevance, coherence, and adequacy of data. The CERQual components reflect similar concerns to the elements included in the GRADE approach for assessing the certainty of evidence on the effectiveness of interventions in previous chapters, however, CERQual considers these issues from a qualitative perspective. The confidence in the evidence for each key finding (theme) was graded as high (it is highly likely that the review finding is a reasonable representation of the phenomenon of interest), moderate (it is likely that the review finding is a reasonable representation of the phenomenon of interest), low (it is possible that the review finding is a reasonable representation of the phenomenon of interest), or very low (it is not clear whether the review finding is a reasonable representation of the phenomenon of interest).

The certainty of the evidence for the key finding “Governance” was moderate in the 16 studies which contributed. The finding was graded as moderate confidence because of moderate concerns regarding methodological limitations, and minor concerns for both coherence and adequacy. The certainty of the evidence for the key finding “RRT Response” was moderate in the 12 studies which contributed. The finding was graded as moderate confidence because of moderate concerns regarding methodological limitations, and minor concerns for both coherence and adequacy. The certainty of the evidence for the key finding “Professional Boundaries” was judged to be high in the 12 studies which contributed. The finding was graded as high confidence because of moderate concerns regarding methodological limitations (particularly in relation to lack of reflexivity) and minor concerns regarding adequacy (rich descriptions of the data were largely were not always provided). These concerns in the two domains were not strong enough to justify downgrading the confidence in the finding. The certainty of the evidence for the key finding “Clinical Experience” was deemed to be high also in the 14 studies which contributed. The finding was graded as high confidence because of moderate concerns regarding methodological limitations (particularly in relation to lack of reflexivity) and minor concerns regarding adequacy (rich descriptions of the data were largely were not always provided). As previously, these concerns were not strong enough to justify downgrading the confidence in the finding. The certainty of the evidence for the key finding “EWS Parameters” was judged to be moderate in the 11 studies which contributed. The finding was graded as moderate confidence because of moderate concerns regarding methodological limitations and coherence and minor concerns regarding adequacy.

Therefore, the overall certainty of the evidence was graded as ‘moderate’. These assessments are summarised in the summary of qualitative findings (SOQF) table

**GRADE CERQual Summary of Qualitative Findings Table**

| **Summary of review finding** | **Studies contributing to the review finding** | **CERQual assessment of confidence in the evidence** | **Explanation of CERQual Assessment** |
| --- | --- | --- | --- |
| ***Governance:***  Participants reported clear, standardised policies and protocols; a good knowledge of the policies, sufficient resources including staff and good communication; and ensuring accountability as facilitators to escalation. Where there was no clear standardised policy or protocol; staff didn’t know the policies; staffing shortages or competing workloads; and lack of accountability or blame, these were reported as barriers to escalation. | 16 studies contributed to this review finding.^(98, 137, 185, 190-198, 200, 201, 203, 204)^ | Moderate confidence  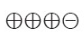 | The finding was graded as moderate confidence because of moderate concerns regarding methodological limitations, and minor concerns for coherence and adequacy. |
| ***RRT Response*:**  The behaviour of the RRT was a key barrier or facilitator to escalation. Where the RRT responded negatively (or not at all) or showed a lack of professionalism to those who made the escalation call, this was reported as a barrier to future escalation by participants. Fear of reprimand by senior staff for making the escalation call or fears of looking stupid were reported barriers to escalation. Where the RRT behaved positively, professionally, collaboratively and made key decisions, using their expertise and provided additional support, this was reported as a facilitator to escalation by participants. | 12 studies contributed to this review finding.^(98, 191-194, 196-200, 202, 203)^ | Moderate confidence  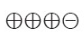 | The finding was graded as moderate confidence because of moderate concerns regarding methodological limitations, and minor concerns for coherence and adequacy. |
| ***Professional Boundaries*:**  The EWS and triggering for help was viewed as a licence to escalate and gave participants increased autonomy. It was also reported to be a bridge across professional boundaries ensuring communication and teamwork across staff levels and a workaround to get a patient seen. Other participants reported including increased conflict among staff (between junior and senior staff) and significant jurisdictional hierarchy as barriers to escalation. | 12 studies contributed to this review finding.^(98, 190-198, 200, 201)^ | High confidence  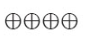 | The finding was graded as high confidence because of moderate concerns regarding methodological limitations and minor concerns regarding adequacy. |
| ***Clinical Experience***:  Clinical confidence to recognise deterioration and confidence in their own ability as well as using one’s clinical judgment were all reported as facilitating factors to escalation by participants. The EWS was also a tool which empowered more junior staff to make the call for help and validated their reason for calling. Some participants reported being unable to recognise deterioration or doubting their own ability to detect deterioration as barriers to making a call for help. Clinical ‘overconfidence’ was also a reported barrier to escalation where staff didn’t call for help due to the belief that they could handle the situation themselves. | 14 studies contributed to this review finding. ^(98, 137, 185, 190-194, 197-201, 203)^ | High confidence  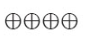 | The finding was graded as high confidence because of moderate concerns regarding methodological limitations and minor concerns regarding adequacy. |
| ***Early Warning Systems Parameters***:  Specific sub-populations (e.g. those with COPD) who resulted in excessive triggering of the EWS and the need for parameter adjustment and modification of the EWS were reported as a deterrent to calling for help by some participants. Others reported that the EWS was an excellent mechanism for triage and ensuring patients received the care they needed as well as a valued tool for detecting deterioration | 11 studies contributed to this review finding. ^(137, 185, 190, 194, 195, 197, 198, 201, 203, 204, 207)^ | Moderate confidence  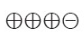 | The finding was graded as moderate confidence because of moderate concerns regarding methodological limitations and coherence and minor concerns regarding adequacy. |

**Key: HCP:** Healthcare Professional; **RRT:** Rapid Response Team; **RRS:** Rapid Response System; **EWS:** Early Warning System; **COPD:** Chronic Obstructive Pulmonary Disease.

**High confidence:** It is highly likely that the review finding is a reasonable representation of the phenomenon of interest

**Moderate confidence:** It is likely that the review finding is a reasonable representation of the phenomenon of interest

**Low confidence:** It is possible that the review finding is a reasonable representation of the phenomenon of interest

**Very low confidence:** It is not clear whether the review finding is a reasonable representation of the phenomenon of interest
